# Differences in Barriers to Healthcare and Discrimination in Healthcare Settings Among Undocumented Immigrants by Deferred Action for Childhood Arrivals (DACA) Status

**DOI:** 10.1007/s10903-022-01346-4

**Published:** 2022-02-28

**Authors:** Rebecca Woofter, May Sudhinaraset

**Affiliations:** Department of Community Health Sciences, University of California Los Angeles Fielding School of Public Health, Los Angeles, CA USA

**Keywords:** Undocumented immigrants, Healthcare, Barriers, Discrimination, Deferred action for childhood arrivals (DACA)

## Abstract

Undocumented immigrants face barriers to and discrimination in healthcare, but those with Deferred Action for Childhood Arrivals (DACA) status may fare better. This analysis uses the cross-sectional BRAVE Study of young undocumented Latinx and Asian immigrants to examine differences in barriers to and discrimination in healthcare by DACA status. A majority of respondents experienced financial, language, and cultural barriers, and up to half experienced documentation status barriers, discrimination when seeking healthcare or by a health provider, and negative experiences related to documentation status. In multivariable analyses, DACA recipients have over 90% lower odds of language and cultural barriers, approximately 80% lower odds of discrimination when seeking healthcare and by a health provider, and approximately 70% lower odds of documentation status barriers and negative experience related to documentation status compared to nonrecipients. These findings indicate that DACA recipients experience fewer barriers to healthcare and discrimination in healthcare compared to nonrecipients.

## Background

Immigrants, particularly undocumented immigrants, face difficulties in accessing healthcare in the United States. Most documented immigrants are unable to access public insurance (e.g., Medicaid) for their first five years of residency, with some exceptions such as refugees and asylees. Documented immigrants can purchase health coverage through the insurance marketplace, and may be eligible for premium tax credits. In contrast, undocumented immigrants are unable to access public insurance or healthcare plans on the insurance marketplace, although some states, such as California, provide coverage for some undocumented immigrants. Even those eligible for insurance may not seek it due to difficulties understanding eligibility or navigating enrollment [1]. This leaves over 40 million immigrants in the US with limited access to health insurance [2]. In addition to difficulty accessing health coverage, many immigrants face additional barriers to healthcare, such as fear of arrest and deportation [3–5]. Among undocumented immigrants, cost of healthcare, lack of health literacy, and transportation issues further impact access to care [5, 6]. These barriers result in low healthcare use and worse self-reported health among this population [6–8].

Immigration policy is a form of structural racism, as it reinforces the social hierarchy and dictates inclusion and exclusion into society [9]. State immigrant policies further include or exclude immigrants, delineating, for example, immigrants’ eligibility for driver’s licenses and in-state college tuition [10]. Inclusive state policies are associated with higher levels of health insurance among Latinx immigrants [11]. Examining structural racism faced by immigrants and the role of intersectional identities is necessary to fully grasp immigrants’ experiences [12].

The Deferred Action for Childhood Arrivals (DACA) program, an inclusive immigrant policy enacted in 2012, may improve access to healthcare for undocumented immigrants. Eligibility requirements for DACA include age and date of arrival in the US, time spent in the US, criminal history, and high school completion or military service. DACA recipients receive temporary protection from deportation and work authorization [13]. Some literature indicates that DACA is associated with improved self-rated health [14, 15], mental health [8, 16–19], sense of belonging [20], and socioeconomic outcomes [17, 21–23]. One paper has shown that DACA recipients experience fewer barriers to healthcare and less discrimination in healthcare than non-recipients, and that these experiences are related to mental health outcomes and gaps in healthcare use [8]. However, the study did not examine the specific barriers to or discrimination in healthcare, or how they may operate independently to influence health outcomes [8]. In another analysis, fewer DACA-eligible respondents than non-eligible respondents reported financial barriers to healthcare, but the authors did not consider other types of healthcare barriers [17].

This paper builds upon existing literature by providing a more precise view of the barriers and experiences undocumented immigrants face in healthcare, and differences by DACA status. Specifically, this paper examines four discrete barriers to healthcare: financial, language, cultural, and documentation status. In addition to financial ability to access care and ability to communicate in a common language with health providers, being understood culturally, with an acknowledgement of how documentation status can impact health, is vital for comprehensive healthcare [24]. When healthcare is not culturally competent, immigrants may feel alienation from and mistrust of the healthcare system, which impairs health outcomes [25]. Additionally, this paper examines three types of discrimination: discrimination when seeking healthcare, discrimination by a health provider, and negative experiences due to documentation status. Experiencing discrimination in the healthcare setting is associated with poor mental and physical health among immigrants [26]. These outcomes individually represent particular elements of the process of receiving healthcare. The first refers to overall experience when seeking healthcare, which may include how inclusive a facility is in its protocols and materials. The second refers to patient-provider interactions. The third highlights experiences in which the facility and/or provider made the patient feel isolated or othered specifically due to their documentation status. Each of these outcomes provides useful information for policy and clinical intervention to improve access to and experiences of healthcare for undocumented immigrants.

## Methods

This analysis uses the BRAVE Study (Building communities, Raising All immigrant Voices for health Equity), which describes the experiences of Latinx and Asian undocumented young adults in California and the impact of DACA on health, social, and economic outcomes [27]. This internet-based survey was open to all Latinx and Asian undocumented immigrant young adults in California. Participants were recruited through social media advertisements and snowball sampling of social networks, and received $20 gift cards for participation. Data were collected between June and August 2017. The survey was overseen by a community advisory board including public health and immigration experts and was approved by the Institutional Review Board at the University of California San Francisco. From the 427 participants, 209 individuals were excluded either because they did not meet eligibility criteria, such as indicating they were born in the US, or provided conflicting responses about their DACA status, such as indicating that they were DACA recipients but entered into the US after the age of 16. This sample was further restricted to include only observations with complete data on all relevant variables in this analysis, resulting in an analytic sample of 203.

### Measures

This analysis uses seven binary outcomes regarding barriers to healthcare and discrimination in healthcare for which participants indicate whether or not they occur. The four questions on barriers to healthcare are: “Do you have difficulties accessing healthcare due to financial barriers? Do you have difficulties accessing healthcare due to language barriers? Do you have difficulties accessing healthcare due to cultural barriers? Does your documentation status prevent you from seeking or accessing healthcare?” The three questions on discrimination in healthcare include: “Do you feel discriminated against when seeking services at a healthcare institution? Do you feel discriminated against by your doctor or other healthcare professional? Have you ever had a negative experience with accessing health care due to your documentation status?”

The main predictor is DACA status, which is captured by asking respondents “Are you currently a DACA recipient?” with options of yes or no. Other relevant sociodemographic independent variables include race/ethnicity (Latinx or Asian), gender (man or woman), level of education (high school or less, some college or 2-year college, and 4-year college degree or above), currently in school (yes or no), currently employed (yes or no), insurance status (yes or no), whether English was spoken at home (yes or no), and age. The majority of Latinx respondents were from Mexico (65%). Asian respondents were most commonly from China (17%), Japan (11%), and Taiwan (11%).

### Analyses

Univariate descriptive statistics were obtained for the sample and compared across DACA status using chi-squared tests and t-tests. Bivariate comparisons across the outcomes by DACA status were made using chi-squared tests. Binary logistic regression models were conducted for each of the outcomes, controlling for all relevant sociodemographic characteristics. Variance inflation factors were assessed for all independent variables in a fully adjusted model, which indicated no concerning levels of multicollinearity. Pseudo-R^2^ values were assessed for each model. Interactions with DACA status and race were explored. All analyses were conducted using STATA 16/SE.

## Results

### Univariate & Bivariate

The sample distribution is shown in Table 1. Approximately one-third of the participants have DACA (34.5%) and are women (37.0%). The sample is nearly evenly split by race (48.3% Latinx and 51.7% Asian). A majority of the sample has some college or a 2-year degree (60.6%) and over half is currently in school (52.2%). Almost 70% of the sample is employed and 71.9% have insurance. Nearly three-quarters speak English at home. The mean age is 23. DACA recipients tend to be Latinx, women, in school, have at least some college education, have insurance, and speak English at home.

Table 2 shows the distribution of each of the outcomes by DACA status. A majority of respondents reported financial, language, and cultural barriers to healthcare. Nearly three-quarters (74%) reported financial barriers, with no statistically significant difference by DACA status. Among DACA recipients, 10% faced language barriers and 36% faced cultural barriers to healthcare, compared with 74% and 90% of those without DACA, respectively. 44% of respondents experienced documentation status barriers, with no statistically significant difference by DACA status.

In terms of discrimination experiences, 42% of respondents faced discrimination when seeking healthcare and 30% faced discrimination by a healthcare provider. Compared with 17% and 9% of DACA recipients who faced discrimination when seeking healthcare and by a healthcare provider, 41% and 55% of non-recipients experienced discrimination, respectively. Approximately half of the participants reported a negative experience when seeking healthcare due to their documentation status, with no statistically significant difference by DACA status.

### Multivariable

Table 3 shows the four binary logistic regression models conducted on barriers to healthcare. As in the bivariate analysis, when controlling for relevant covariates, DACA recipients’ odds of financial barriers did not statistically significantly differ from non-recipients (aOR 0.34, 95% CI 0.11, 1.03). Controlling for relevant covariates, DACA recipients had 92% lower odds of language barriers (aOR 0.08, 95% CI 0.03, 0.24) and 91% lower odds of cultural barriers (aOR 0.09, 95% CI 0.03, 0.23) compared to non-recipients. Although DACA status was not statistically significant in the bivariate test with documentation status barriers, when controlling for relevant covariates, DACA recipients had 71% lower odds of documentation status barriers (aOR 0.29, 95% CI 0.11, 0.76) compared to non-recipients. Notably, Asian individuals had over six times higher odds of experiencing language barriers than Latinx individuals (aOR 6.60, 95% CI 2.16, 20.17). These models explained 23%, 43%, 32%, and 25% of the variance in each outcome, respectively, based on the pseudo-R^2^.

Table 4 shows the three binary logistic regression models conducted on discrimination measures. Controlling for relevant covariates, DACA recipients had 79% lower odds of experiencing discrimination when seeking healthcare (aOR 0.21, 95% CI 0.08, 0.56) and 82% lower odds of experiencing discrimination by a healthcare provider (aOR 0.18, 95% CI 0.06, 0.58) compared to non-recipients. Although DACA status was not statistically significant in bivariate analysis for having a negative experience based on documentation status, when controlling for relevant covariates, DACA recipients had 73% lower odds of a negative experience due to documentation status (aOR 0.27, 95% CI 0.11, 0.68) compared to non-recipients. Notably, women had nearly four times higher odds of reporting discrimination by a health provider than men (aOR 3.93, 95% CI 1.44, 10.74). These models explained 25%, 33%, and 19% of the variance in each outcome, respectively, based on the pseudo-R^2^.

An exploratory analysis was conducted to assess the combined impact of DACA status and race (data not shown). These indicated that Asian DACA recipients had higher odds of experiencing documentation status barriers to healthcare, discrimination by a health provider, and a negative experience due to documentation status compared to Latinx DACA recipients. However, the limited sample size of Asian DACA recipients (n = 18) resulted in wide confidence intervals, despite the large odds ratios and small p-values (p = 0.00).

## Discussion

This study is one of the first to examine specific barriers and discrimination undocumented immigrants face in healthcare by DACA status. In this sample, levels of barriers to healthcare and discrimination in healthcare were high. The vast majority of undocumented immigrants face financial barriers to healthcare, and even those with DACA struggle to afford healthcare. A lower proportion of DACA recipients than non-recipients reported experiencing all barriers and forms of discrimination. When controlling for relevant covariates, DACA recipients had statistically significantly lower odds of reporting language, cultural, and documentation status barriers, and experiences of discrimination when seeking healthcare and by a healthcare provider, and having negative experiences when seeking healthcare due to documentation status, compared to non-recipients.

Given the sociodemographic differences between DACA recipients and non-recipients, those who successfully navigate the DACA process may be fundamentally different from those who do not. The process of applying for DACA requires a fee of $495 and a legal form with supporting documentation, and must be renewed every 2 years [13]. Individuals possessing the skills and funds for this process may, as a result of that privilege, have better healthcare experiences than those who do not, even if they did not have DACA status. This cross-sectional study is not able to assess any causal effect of DACA, but highlights the disparity in healthcare access and experiences by DACA status.

This study is unique in including both Latinx and Asian undocumented immigrants. Although most immigrants in California were born in Latin America, the majority of recent arrivals are from Asia, indicating the need to center Asian populations in immigrant studies [28]. Nationally, 94% of DACA recipients are from Latin America, with just 3% from Asia, highlighting a disparity in obtaining DACA [29]. In this analysis, Asian individuals had significantly higher odds of experiencing language barriers compared to Latinx individuals. Most literature on undocumented immigrants is limited to Latinx immigrants, yet studies suggest that Asian undocumented immigrants may face unique challenges, including cultural struggles and the model minority myth [30, 31]. The sample was not powered to detect differences in healthcare experiences by both DACA status and race, nor was it sufficiently large to examine within-race differences by ethnicity. Future research should examine these subgroups.

This study confirms and expands the findings of previous literature. Using the National Health Interview Survey, one study found that after 2012, the DACA-eligible population experienced a 21% decrease in likelihood to delay care due to finances and a 5% decrease in the inability to seek care due to finances [17]. The present study, which directly assesses rather than estimates DACA status, did not detect a statistically significant difference in financial barriers to healthcare by DACA status, but provides estimates of differences in language, cultural, and documentation status barriers. A qualitative study with Asian and Pacific Islander undocumented immigrants found low levels of healthcare access and use, with some improvement after receiving DACA [30]. A qualitative study with Latinx DACA-eligible immigrants similarly found low levels of healthcare access and use, low health literacy, and high financial barriers and fear of seeking healthcare. Those with DACA indicated that it improved their access to care, but did not fully mitigate barriers [32]. The present study quantitatively affirms the barriers suggested in these qualitative studies, demonstrating specific advantages in healthcare experience among DACA recipients compared to non-recipients. Additionally, this study confirms that regardless of DACA status, undocumented immigrants face many barriers to healthcare.

This study also provides important findings about discrimination faced by undocumented immigrants. Existing literature using the California Health Interview Survey has demonstrated that people of color and immigrants report more discrimination in healthcare settings than their white and US-born counterparts [33, 34]. By separating experiences of discrimination when seeking healthcare broadly and by a health provider particularly in this study, more nuanced patterns emerge. First, a larger proportion of respondents report experiencing discrimination when seeking healthcare than by a healthcare provider, and more still report a negative experience due to documentation status. This indicates that while patient-provider interactions are important, the process of accessing healthcare (e.g., securing an appointment, completing paperwork) is also salient to immigrants’ experiences in healthcare. Ensuring that all staff and materials are inclusive of undocumented immigrants may improve quality of healthcare for this population. Additionally, women have significantly higher odds of reporting discrimination by a healthcare provider compared to men, but not when seeking healthcare broadly. This suggests that in addition to race and documentation status, gender may further impact patient-provider interactions. Literature suggests that Black and Latina women experience gendered racism, particularly in pregnancy- or family planning-related healthcare [35, 36]. A qualitative study of Hispanic immigrant women found that a majority experienced discrimination by a health provider based on stereotypes of their race, immigration status, and language ability [37]. These intersections should be further explored, and particularly investigated among Asian women.

This study has several limitations. First, this study includes a small sample, and leaves some small cells in analyses. However, no cells had fewer than 5 individuals, and sequential model-building with each covariate showed stable estimates of the effect of DACA for each outcome. Second, DACA recipients and non-recipients in this sample differ across sociodemographic characteristics. Although multivariable models control for these differences, unmeasured confounding may occur on characteristics such as income and social capital. Third, this is a convenience sample in California, which may not be representative of all undocumented immigrants in California or nationally. Indeed, California is an immigrant-friendly state, and studies demonstrate that experiences of DACA recipients differ based on state of residence [38]. Given that California provides many undocumented young adults with access to healthcare via MediCal [1], this study may actually underestimate the differences in healthcare access and experiences by DACA. Finally, the outcomes in this analysis are self-reported, and individuals who face the same circumstances may not all identify their experiences the same way.

Despite these limitations, this study has several strengths and provides valuable new insight to the literature on access to and experiences in healthcare among undocumented immigrants and differences by DACA status. This is one of few quantitative analyses with undocumented immigrants who directly report their DACA status, as opposed to estimations of DACA-eligible individuals. This is particularly important when studying Asian undocumented immigrants, as estimation techniques are prone to bias [39]. It also includes several measures for barriers to healthcare and types of discrimination, which allows for nuance in understanding healthcare access and experiences. Importantly, it includes both Latinx and Asian individuals, which allows for cross-racial comparisons. Finally, given that all participants live in California, there is no concern that state-level policies are causing heterogeneity in experiences. Indeed, given that 25% of all immigrants in the US reside in California [28], it is an ideal setting for studying this population.

Undocumented immigrants face several barriers to accessing healthcare and discrimination when they do seek care, though less commonly among DACA recipients than non-recipients. The Biden administration is considering providing a path to citizenship for many undocumented immigrants [40] and others have described additional steps the administration can take to improve immigrant health [41]. Inclusive policies and programs should provide avenues to healthcare regardless of documentation status in order to address financial, language, cultural, and documentation status barriers to healthcare. In particular, expanding health insurance eligibility regardless of documentation status and funding low-cost community clinics may help reduce financial barriers, and requiring translators in healthcare offices, particularly for Asian languages, may reduce language barriers. Health providers have a responsibility to ensure they and their staff are providing patients with respectful and culturally competent care, with sensitivity to the needs of undocumented immigrants.

**Table 1:** Distribution of sample characteristics by DACA status (n = 203)

	Total (n, %) or mean (sd)	DACA Recipients (n, %) or mean (sd)	Non-DACA Recipients (n, %) or mean (sd)	P-value of chi-squared tests or t-tests
DACA				
No DACA	133 (65.5%)	–	–	
Have DACA	70 (34.5%)	–	–	
Race				0.000
Latinx	98 (48.3%)	58 (82.9%)	40 (30.1%)	
Asian	105 (51.7%)	12 (17.1%)	93 (69.9%)	
Gender				0.000
Men	128 (63.1%)	26 (37.1%)	102 (76.7%)	
Women	75 (37.0%)	44 (62.9%)	31 (23.3%)	
Education				0.000
High school or less	47 (23.2%)	13 (18.6%)	34 (25.6%)	
Some college/2-year	123 (60.6%)	35 (50.0%)	88 (66.2%)	
4-year College degree +	33 (16.3%)	22 (31.4%)	11 (8.3%)	
In school				0.000
No	97 (47.8%)	17 (24.3%)	80 (60.2%)	
Yes	106 (52.2%)	53 (75.7%)	53 (40.0%)	
Employed				0.93
No	63 (31.0%)	22 (31.4%)	41 (30.8%)	
Yes	140 (69.0%)	48 (68.6%)	92 (69.2%)	
Have insurance				0.06
No	57 (28.1%)	14 (20.0%	43 (32.3%)	
Yes	146 (71.9%)	56 (80.0%)	90 (67.7%)	
Speak english at home				0.003
No	52 (25.6%)	9 (12.9%)	43 (32.3%)	
Yes	151 (74.4%)	61 (87.1%)	90 (67.7%)	
Age	23 (3.4)	23 (3.4)	23 (3.4)	0.19

**Table 2:** Distribution of outcomes by DACA status (n = 203)

	Total (n, %) or mean (sd)	DACA recipients (n, %) or mean (sd)	Non-DACA recipients (n, %) or mean (sd)	p-Value of chi-squared tests
Experience this type of barriers				
Financial	151 (74.4%)	51 (72.9%)	100 (75.2%)	0.72
Language	106 (52.2%)	7 (10.0%)	99 (74.4%)	0.000
Cultural	144 (70.9%)	25 (35.7%)	119 (89.5%)	0.000
Documentation	89 (43.8%)	28 (40.0%)	61 (45.9%)	0.42
Experience discrimination in healthcare settings				
When seeking services	85 (41.9%)	12 (17.1%)	73 (54.9%)	0.000
By health provider	61 (30.1%)	6 (8.6%)	55 (41.4%)	0.000
Negative experience due to documentation status	101 (49.8%)	30 (42.9%)	71 (53.4%)	0.154

**Table 3:** Multivariable binary logistic regression models estimating barriers to seeking healthcare by DACA status controlling for relevant covariates (n = 203)

	Financial (aOR, 95% CI)	Language (aOR, 95% CI)	Cultural (aOR, 95% CI)	Documentation (aOR, 95% CI)
DACA				
No DACA	Ref	Ref	Ref	Ref
Have DACA	0.34 (0.11, 1.03)	0.08 (0.03, 0.24)***	0.09 (0.03, 0.23)***	0.29 (0.11, 0.76)*
Race				
Latinx	Ref	Ref	Ref	Ref
Asian	0.37 (0.12, 1.18)	6.60 (2.16, 20.17)**	1.79 (0.64, 5.01)	0.51 (0.20, 1.28)
Gender				
Men	Ref	Ref	Ref	Ref
Women	0.92 (0.39, 2.21)	2.39 (0.99, 5.75)	1.84 (0.77, 4.39)	0.62 (0.27, 1.42)
Education				
High School or less	Ref	Ref	Ref	Ref
Some college/2-year	1.11 (0.41, 3.03)	1.95 (0.73, 5.21)	1.77 (0.66, 4.80)	1.58 (0.64, 3.90)
4-year College degree +	0.59 (0.16, 2.15)	1.19 (0.27, 5.36)	2.86 (0.72, 11.33)	0.76 (0.22, 2.57)
In school				
No	Ref	Ref	Ref	Ref
Yes	3.73 (1.62, 8.61)**	1.49 (0.57, 3.87)	1.77 (0.67, 4.80)	4.64 (1.98, 10.86)***
Employed				
No	Ref	Ref	Ref	Ref
Yes	1.11 (0.49, 2.50)	0.58 (0.22, 1.49)	1.71 (0.71, 4.12)	3.38 (1.49, 7.68)**
Have insurance				
No	Ref	Ref	Ref	Ref
Yes	0.10 (0.03, 0.40)**	0.30 (0.10, 0.88)*	0.64 (0.24, 1.68)	0.29 (0.12, 0.71)**
Speak snglish at home				
No	Ref	Ref	Ref	Ref
Yes	2.53 (1.08, 5.92)*	1.08 (0.37, 3.15)	1.53 (0.53, 4.41)	2.95 (1.17, 7.48)*
Age	1.03 (0.91, 1.18)	1.18 (1.01, 1.37)*	0.99 (0.87, 1.14)	1.11 (0.98, 1.27)

*p < 0.05, **p < 0.01, ***p < 0.001

**Table 4:** Multivariable binary logistic regression models estimating discrimination in healthcare by DACA status controlling for relevant covariates (n = 203)

	Discrimination when seeking healthcare (aOR, 95% CI)	Discrimination by health provider (aOR, 95% CI)	Negative healthcare experience due to documentation status (aOR, 95% CI)
DACA			
No DACA	Ref	Ref	Ref
Have DACA	0.21 (0.08, 0.56)**	0.18 (0.06, 0.58)**	0.27 (0.11, 0.68)**
Race			
Latinx	Ref	Ref	Ref
Asian	1.78 (0.64, 4.90)	1.71 (0.53, 5.55)	0.47 (0.19, 1.17)
Gender			
Men	Ref	Ref	Ref
Women	1.20 (0.54, 2.66)	3.93 (1.44, 10.74)**	1.10 (0.51, 2.34)
Education			
High School or less	Ref	Ref	Ref
Some college/2-year	2.14 (0.87, 5.26)	2.51 (0.91, 6.91)	1.98 (0.83, 4.71)
4-year College degree +	0.94 (0.26, 3.38)	0.19 (4.11)	2.71 (0.84, 8.81)
In School			
No	Ref	Ref	Ref
Yes	1.81 (0.79, 4.14)	1.81 (0.73, 4.50)	2.34 (1.09, 5.06)*
Employed			
No	Ref	Ref	Ref
Yes	1.90 (0.94, 4.30)	6.70 (2.23, 20.12)**	1.94 (0.93, 4.04)
Have insurance			
No	Ref	Ref	Ref
Yes	0.37 (0.15, 0.90)*	0.37 (0.13, 1.05)	0.40 (0.18, 0.92)*
Speak english at home			
No	Ref	Ref	Ref
Yes	1.24 (0.52, 2.95)	2.51 (0.83, 7.57)	2.33 (1.02, 5.31)*
Age	1.27 (1.10, 1.47)**	1.07 (0.93, 1.24)	1.15 (1.01, 1.30)*

*p < 0.05, **p < 0.01
